# Editorial: Women in environmental microbiomes

**DOI:** 10.3389/frmbi.2025.1537069

**Published:** 2025-01-24

**Authors:** Rebecca C. Mueller, Catherine A. Gehring

**Affiliations:** ^1^ Invasive Species and Pollinator Health Unit, Western Regional Research Center, United States Department of Agriculture (USDA) Agricultural Research Service, Albany, CA, United States; ^2^ Department of Biological Sciences and Center for Adaptable Western Landscapes, Northern Arizona University, Flagstaff, AZ, United States

**Keywords:** industrial waste, climate change, human health, host traits, agriculture, bioremediation

Diversity and productivity scale across multiple systems, from ecosystems to workplaces (Saxena, 2014, https://futurumcareers.com/why-is-diversity-important-for-productivity). The productivity, novelty, and impact of gender-diverse research groups exceed those with less equity (Sarabi and Smith, 2023), indicating that gender biases that exclude women from STEM careers are limiting the scope of scientific breakthroughs from some of the best potential scientists. Multiple lines of evidence suggest that female scientists are subjected to discrimination during their careers. Despite progress in earning advanced degrees in STEM, women remain under-represented in higher level career positions (Ross et al., 2022; Llorens et al., 2021), particularly tenure-track positions in academia (Casad et al., 2021). Women are less likely to be credited in scientific papers for research done in teams, particularly in high impact research (Ross et al., 2022) and receive more negative reviews both when applying for intramural grants (Witteman et al., 2019) and submitting manuscripts for publication (Hagan et al., 2020). Likely due to societal expectations, female scientists show less willingness to self-promote compared to male scientists. Relative to men, women are less likely to cite their prior work in subsequent publications (King et al., 2017) and undersell their abilities in self-evaluations when questions are focused on tasks generally perceived as male-type (Exley and Kessler, 2022). Biases against female scientists are often perpetuated by women. Female faculty showed similar bias against women as male faculty during the hiring process (Moss-Racusin et al., 2012), emphasizing the need for mentorship activities that reduce biases based on gender to encourage women’s participation in STEM at all career stages (Stout et al., 2011). Despite these limitations, signs of progress in gender equity in STEM are emerging; in the last decades, representation for female authors in scientific publications (Huang et al., 2020) and prestigious honorary societies such as the National Academy of Sciences (Card et al., 2023) have increased, along with increasing willingness to hire female faculty (Williams and Ceci, 2015).

This Research Topic, titled “Women in Environmental Microbiomes”, is part of a series of Research Topics launched by Frontiers to celebrate International Women’s Day (March 8 each year) and includes nine publications with a female researcher as the first author or the corresponding author; more than 40 female authors contributed to these studies. The featured papers illustrate how women scientists, from students to leaders of well-established research labs, are furthering the study of microbiomes. The goal of *Frontiers in Microbiomes* is to “advance our understanding of how microbiomes generate positive or negative outcomes for their hosts and environments”. This goal reflects increasing recognition that a diverse suite of microbes occurs in every environment where they influence processes at every level of biological organization. The term “microbiome” is now often recognized by the public because of pioneering research on humans that began with microbial community characterization, expanded to the mechanisms underpinning host-microbiome interactions, identifying linkages between a diversity of human conditions and microbial communities that led to novel treatments (Rackaityte and Lynch, 2020). Some of the earliest work investigating connections between consortia of microbes and human health was led by a woman, Dr. Abigail Salyers, who successfully demonstrated the importance of microbes other than model taxa that were the focus of previous research (Whitaker and Barton, 2018). The rapidly growing study of the plant microbiome including its metabolite production also is leading to new understanding and applications, particularly in agriculture (Compant et al., 2025; Saikkonen et al., 2020). Expanded development and deployment of ‘omics tools and world-wide collaboration has begun to provide a synthetic understanding of the forces that shape the microbiome of the planet (Thompson et al., 2017).

Our view of the elements of the microbiome and the techniques available to study it also have expanded over the past two decades, changes that are illustrated in this Research Topic. While early microbiome research focused on bacteria, the field now investigates and acknowledges the importance of consortia of viruses, archaea, fungi and protists, often in combination. For example, two studies in this special feature explore how microbiome analysis at different temporal or spatial scales within a common system yielded insights into broader relationships. Sacharow et al. used amplicon sequencing to explore patterns of Cercozoan protist diversity in spring barley (*Hordeum vulgare*) comparing leaf, root and soil compartments at two different developmental stages. Protists are arguably the least studied members of the microbiome though their complex roles in the rhizosophere and plant health are increasingly documented (Xiong et al., 2020; Bahroun et al., 2021). Sacharow et al. found strong differences in protist communities among leaves, roots and soils but few differences related to plant developmental stage. Both sampling location and time were important to the bacterial and viral communities of the River Erpe examined by Rodriguez-Ramos et al. Repeated sampling and metagenomic analysis of surface and porewater compartments showed distinct communities associated with each of the compartments and identified highly consistent viral communities over time in surface waters but not in porewater (Rodriguez-Ramos et al.). These studies illustrate the value of complex sampling designs that explore microbial diversity across multiple gradients simultaneously.

The effect of host genetics and host traits on microbiome composition and function has been an important area of study, particularly in human health and agriculture. Two papers in this Research Topic highlight the value of these types of studies while also exploring relevant societal issues - our poor understanding of women’s health issues and the importance of assessing changes to the soil microbiome following the planting of transgenic crops. Zheng et al. used shotgun sequencing of the bacteria and virus/phage communities of the vaginal microbiome of women with and without Polycystic Ovarian Syndrome (PCOS) and obesity to show that vaginal dysbiosis was associated with a decrease in phages alongside increased bacterial diversity (Zheng et al.). In contrast, the vaginal microbiomes of women without PCOS and who were not obese were similar to one another and less diverse. This study advances our understanding of an important health concern for many women as PCOS is a leading cause of infertility globally, yet remains poorly diagnosed and understood (WHO - https://www.who.int/news-room/fact-sheets/detail/polycystic-ovary-syndrome). In fact, many diseases that affect women are poorly understood potentially due to underfunding of research on those diseases relative to the death and disability they cause (Smith, 2023). Similarly, generating sufficient food to feed a growing global population requires an understanding of the potential consequences of leveraging emerging technologies to boost production. As we increasingly use genetic tools to alter the traits of important agricultural crops, assessment of changes in microbiome composition and function are critical to ensuring the safety of these transgenic crops for large scale planting. As of July 2024, > 90% of U.S. corn, upland cotton, and soybeans were produced using genetically engineered varieties, with genetic engineering primarily focused on herbicide tolerance and insect resistance (https://www.ers.usda.gov/data-products/adoption-of-genetically-engineered-crops-in-the-united-states/recent-trends-in-ge-adoption/). One of several concerns of this widespread use of transgenic crops is an inadvertent and potentially detrimental impact on non-target species that could alter soil biodiversity and ecosystem function. Yang et al. examined the effect of a different type of genetic modification of cotton, the insertion of *ScALDH21* from a desiccation-tolerant moss, on soil bacterial and fungal communities. They observed that the diversity and dominant fungal and bacterial communities at both the phylum and genus level were similar between transgenic and non-transgenic genotypes. The differences they observed in microbial communities were associated with soil properties rather than plant genotype. Although these two studies arrive at different answers regarding host traits, they both carefully document a range of possible influences on microbiome composition to strengthen their conclusions.

The importance of the microbiome in accurately predicting how ecosystems respond to both biotic and abiotic perturbations is particularly important now as rapid environmental changes are occurring in many areas of the world. For example, in 2024, South America had the second driest October on record while North America had the 12^th^ driest. Dry conditions were accompanied by record high temperatures that exacerbated dry conditions resulting in severe drought (NOAA Global Drought Monitor https://gdis-noaa.hub.arcgis.com/pages/drought-monitoring). Goemann et al. combined amplicon sequencing of archaea, bacteria and fungi with metabolomics of root exudates to examine the impact of differing levels of drought stress on the rhizobiome of a widespread prairie grass, *Bouteloua gracilis*, identifying drought-responsive clades in all microbial groups, along with metabolites that were more abundant in drought-stressed plants. Natural disturbances such as volcanic eruptions also can alter microbial communities to varying degrees depending on the intensity of disturbance. Maltz et al. used amplicon sequencing to document the responses of fungi, arbuscular mycorrhizal fungi, archaea and bacteria to different landscapes affected by the eruption of Mount St. Helens, finding that old growth and clearcut forests differed in fungal community composition, but not diversity while areas of the pumice plain colonized by lupine had higher diversity of particular guilds of arbuscular mycorrhizal fungi in plots with historic access to pocket gophers. Studies on the soil microbiome also focus increasingly on restoration of microbes that have been reduced or lost due to disturbance. Markovchick et al. used field experiments to examine the legacy effects of an invasive plant, mediated through soil modifications, to subsequent success of native species. They found that active restoration of altered microbial communities substantially improved native plant survival. Understanding microbiome responses and how they feed back to affect native vegetation is critically important to predicting future plant distributions and to implementing restoration following disturbance (Coban et al., 2022).

Many industrial applications depend on microbial metabolism, including wastewater treatment and production of biofuels, but these communities often have been treated as a “black box”, with little understanding of the organisms or functional pathways that drive these processes. Harnessing the metabolic potential of the microbial community would be best applied by identifying the organisms best suited for these services. Pharmaceutical residues are now found worldwide in aquatic and terrestrial ecosystems (Patel et al., 2019). Due to their harmful effects on flora and fauna, identifying efficient mechanisms for removal from wastewater is critical. Bodle et al. examined the role of microbial activity in the degradation of pharmaceuticals commonly released into wastewater streams, and found initial degradation declined along with the abundance of key bacterial and archaeal families. Similarly, fiber byproducts from agriculture and forestry are waste products that can be used to generate biofuels, chemicals and materials. Wong et al. used metagenomic sequencing of communities from an industrial anaerobic digester to characterize carbohydrate active enzymes and found that a small subset of the community participated in lignocellulose degradation, including poorly characterized lineages. Their results suggest there is still unlocked potential of microbes that would improve the transformation efficiency of these byproducts. A better understanding of the taxonomy and function of communities in biotechnology applications will likely improve efficiencies and outcomes, demonstrating the value of a mechanistic understanding of microbiomes to refine their services for human well-being.

We thank the authors, reviewers and subject editors who contributed to this Research Topic and helped highlight the important contributions of women across the globe to the field of microbiome research.
